# Evidence for Recombinant GRP78, CALR, PDIA3 and GPI as Mediators of Genetic Instability in Human CD34+ Cells

**DOI:** 10.3390/cancers14122883

**Published:** 2022-06-11

**Authors:** Alice Fabarius, Vanessa Samra, Oliver Drews, Handan Mörz, Miriam Bierbaum, Ali Darwich, Christel Weiss, Susanne Brendel, Helga Kleiner, Wolfgang Seifarth, Wolfgang Greffrath, Wolf-Karsten Hofmann, Clemens A. Schmitt, Henning D. Popp

**Affiliations:** 1 Department of Hematology and Oncology, Medical Faculty Mannheim, Heidelberg University, 68167 Mannheim, Germany; alice.fabarius@medma.uni-heidelberg.de (A.F.); vanessa.kohl@medma.uni-heidelberg.de (V.S.); susanne.brendel@medma.uni-heidelberg.de (S.B.); helga.kleiner@medma.uni-heidelberg.de (H.K.); wolfgang.seifarth@medma.uni-heidelberg.de (W.S.); w.k.hofmann@medma.uni-heidelberg.de (W.-K.H.); 2 Biomedical Mass Spectrometry, Center for Medical Research, Johannes Kepler University, 4020 Linz, Austria; oliver.drews@jku.at; 3 Department of Neurophysiology, Medical Faculty Mannheim, Heidelberg University, 68167 Mannheim, Germany; handan.moerz@medma.uni-heidelberg.de (H.M.); wolfgang.greffrath@medma.uni-heidelberg.de (W.G.); 4 Department of Radiation Oncology, Medical Faculty Mannheim, Heidelberg University, 68167 Mannheim, Germany; miriam.bierbaum@medma.uni-heidelberg.de; 5 Department of Orthopedics and Trauma Surgery, Medical Faculty Mannheim, Heidelberg University, 68167 Mannheim, Germany; ali.darwich@umm.de; 6 Department of Medical Statistics and Biomathematics, Medical Faculty Mannheim, Heidelberg University, 68167 Mannheim, Germany; christel.weiss@medma.uni-heidelberg.de; 7 Department of Hematology and Oncology, Kepler University Hospital, Johannes Kepler University, 4020 Linz, Austria; clemens.schmitt@kepleruniklinikum.at; 8 Medical Department, Division of Hematology, Oncology and Tumor Immunology, Campus Virchow-Klinikum, Charité—Universitätsmedizin Berlin, Corporate Member of Freie Universität Berlin and Humboldt-Universität zu Berlin, 13353 Berlin, Germany; 9 Max-Delbrück-Center for Molecular Medicine, Helmholtz Association, 13125 Berlin, Germany

**Keywords:** ionizing irradiation, mesenchymal stromal cells, genotoxic signaling, GRP78, CALR, PDIA3, GPI, CD34+ cells, genetic instability

## Abstract

**Simple Summary:**

Factors secreted from irradiated mesenchymal stromal cells (MSC) may exert genotoxic effects in human CD34+ cells, thereby potentially contributing to the pathogenesis of hematologic disorders such as leukemias. In a proteomics approach, we recently identified four key proteins in the secretome of X-ray-irradiated MSC, among them the three chaperones GRP78, CALR, and PDIA3, and one glycolytic enzyme GPI. Here, we demonstrate that recombinant GRP78, CALR, PDIA3 and GPI induce significant levels of genetic instability in human CD34+ cells. Our data suggest that GRP78, CALR, PDIA3 and GPI released from irradiated MSC act as mediators of genetic instability in human CD34+ cells with potential implications for radiation-induced hematologic disorders.

**Abstract:**

Soluble factors released from irradiated human mesenchymal stromal cells (MSC) may induce genetic instability in human CD34+ cells, potentially mediating hematologic disorders. Recently, we identified four key proteins in the secretome of X-ray-irradiated MSC, among them three endoplasmic reticulum proteins, the 78 kDa glucose-related protein (GRP78), calreticulin (CALR), and protein disulfide-isomerase A3 (PDIA3), as well as the glycolytic enzyme glucose-6-phosphate isomerase (GPI). Here, we demonstrate that exposition of CD34+ cells to recombinant GRP78, CALR, PDIA3 and GPI induces substantial genetic instability. Increased numbers of γH2AX foci (*p* < 0.0001), centrosome anomalies (*p* = 0.1000) and aberrant metaphases (*p* = 0.0022) were detected in CD34+ cells upon incubation with these factors. Specifically, γH2AX foci were found to be induced 4–5-fold in response to any individual of the four factors, and centrosome anomalies by 3–4 fold compared to control medium, which contained none of the recombinant proteins. Aberrant metaphases, not seen in the context of control medium, were detected to a similar extent than centrosome anomalies across the four factors. Notably, the strongest effects were observed when all four factors were collectively provided. In summary, our data suggest that specific components of the secretome from irradiated MSC act as mediators of genetic instability in CD34+ cells, thereby possibly contributing to the pathogenesis of radiation-induced hematologic disorders beyond direct radiation-evoked DNA strand breaks.

## 1. Introduction

Specific factors released from irradiated human mesenchymal stromal cells (MSC) may induce genetic instability in non-irradiated human CD34+ cells potentially mediating hematologic disorders. Such disorders may comprise radiation-induced secondary myeloid neoplasias, acquired aplastic anemia or graft failure of hematopoietic stem cell transplants after total body irradiation [1]. In general, the effects in non-irradiated cells caused by signal transmission from irradiated cells are termed non-targeted effects (NTE) and are, at the level of DNA strand breaks, resembling the DNA damage in directly irradiated cells [2,3,4]. NTE have been demonstrated previously in mouse hematopoietic stem and progenitor cells (HSPC) [5,6] and recently by ourselves in human HSPC [7,8].

In general, diverse mediators such as calcium fluxes [9,10], mitochondrial metabolites [9,11], nitric oxide (NO) [12,13], reactive oxygen species (ROS) [13,14], IL-1 beta, IL-8, TNF-alpha [15], TGFbeta-1 [16,17], cathepsin B [18], NF-kappa B [19], MAP kinases [20], microRNA, mitochondrial DNA and cell-free chromatin [21,22,23] have been shown to be involved in DNA damage signaling between irradiated and non-irradiated cells. Depending on their physico-chemical properties, transmission may occur by diverse mechanisms such as diffusion, through gap junctions, by exocytosis or secretion in exosomes/exosome-like vesicles [21,22,24].

Using nanoscale liquid chromatography coupled to tandem mass spectrometry we recently investigated genotoxic signals released from irradiated MSC, over their transmission in MSC conditioned medium, to their transduction in CD34+ cells exposed to MSC conditioned medium [7]. Fractionation experiments revealed that the genotoxic mediators belong to the 10–100 kDa fraction of MSC conditioned medium and that these mediators are heat-sensitive [8]. Specifically, three proteins of the endoplasmic reticulum (ER) and a glycolytic/gluconeogenic protein were identified as key mediators in the MSC conditioned medium: 78 kDa glucose-related protein (GRP78), calreticulin (CALR), protein disulfide-isomerase A3 (PDIA3) and glucose-6-phosphate isomerase (GPI).

GRP78, CALR and PDIA3 are chaperones that antagonize ER stress, which is characterized by an increase in un-/misfolded proteins in the ER lumen. When ER stress occurs, GRP78 dissociates from the luminal domains of IRE1, PERK and ATF6 which mediates activation of the unfolded protein response [25] and the ER-associated protein degradation pathway [26]. When ER stress continues, GRP78 may be transported via the Golgi apparatus and secretory granules to the plasma membrane and may be released in the extracellular space [27]. Distinct solid tumor cells such as PC-3 prostate and HRT-18 colon carcinoma cells are able to secrete high amounts of GRP78 into the tumor microenvironment [28]. Secreted and cell surface GRP78 might bind to Cripto-1, a multifaceted developmental oncoprotein located on the cell surface. Cripto-1 is involved in embryo- and carcinogenesis via MAPK/ERK, PI3K/Akt and Smad2/3 signaling [29]. Cell surface GRP78 forms a complex with Cripto-1 and functions as a necessary mediator of Cripto-1 signaling in human tumor and embryonic stem cells [29]. In addition, GRP78 is overexpressed on the cell surface of acute myeloid leukemia (AML) and different leukemic cell lines [30] but not on HSPC making it an attractive target for CAR T cell therapy [31].

CALR is a component of the calreticulin/calnexin cycle in the ER and ensures regular folding of newly formed glycoproteins [32]. CALR contributes to intracellular Ca^2+^ homeostasis by buffering Ca^2+^ in the ER. In addition, CALR is involved in danger signaling when exposed on the cell surface as a consequence of activation of the integrated stress response [33]. Moreover, *CALR* mutations occur in *JAK2/MPL* wild-type myeloproliferative neoplasms and are encoded in about 85% by type-1 (52 base pair deletion) and type-2 mutations (5 base pair insertion) [34]. Type 1/2 *CALR* mutations result in loss of the KDEL sequence, which retains CALR in the ER. Consequently, mutated CALR is transported from the ER to the cell surface and induces constitutive activation of MPL oncogenic signaling via JAK2 and MAPKs.

PDIA3 is a disulfide isomerase, which catalyzes the formation/isomerization and reduction/oxidation of disulfide bonds [35]. According to its enzymatic function PDIA3 is necessary for the regular folding of nascent glycoproteins [36]. PDIA3 is located in other cellular compartments than the ER. In the nucleus PDIA3 interacts with different proteins such as STAT3, Ape/Ref1, Ku80 and different DNA sequences and structures. Here, PDIA3 is involved in gene regulation, DNA repair and transcriptional factor reduction [37]. Further, PDIA3 is present in mitochondria-associated membranes and interferes with mitochondrial bioenergetic function. In addition, PDIA3 alters STAT3 signaling in the cytosol [38] and secreted PDIA3 may facilitate activation of metalloproteases and integrins at the surface of adjacent cells which may promote carcinogenesis [39]. Moreover, PDIA3 levels are elevated in AML bone marrow cells [40].

GPI catalyzes the conversion of glucose-6-phosphate to fructose-6-phosphate during glycolysis/gluconeogenesis; however, aside from its metabolic function, GPI may act as a tumor-secreted cytokine as well and stimulate tumor cell motility and angiogenesis [41]. In this context, GPI is termed autocrine motility factor (AMF). GPI/AMF is the ligand of the cell surface AMF receptor, which activates RhoA/Rac1 [42] MAPK/ERK [43] and PI3K/AKT signaling pathways [44]. Further, overexpression of GPI/AMF inducesPI3K/AKT-mediated transformation and survival of NIH/3T3 mouse fibroblasts [44], whereas exogenous GPI/AMF protects against ER stress in COS7 cells [45].

Since GRP78, CALR, PDIA3 and GPI are key factors with oncogenic potential in MSC conditioned medium, we aimed here at further investigating oncogenic effects of these proteins on CD34+ cells. For this purpose, healthy human CD34+ cells were exposed to recombinant GRP78, CALR, PDIA3 and GPI proteins and genotoxic effects were analyzed.

## 2. Materials and Methods

### 2.1. Preparation of Femoral Heads

This study was approved by the Ethics Committee II, Medical Faculty Mannheim, Heidelberg University (no. 2019-1128N). Procedures were performed in accordance with the local ethical standards and the principles of the 1964 Helsinki Declaration and its later amendments. Written informed consent was obtained from all study participants. Femoral heads were collected from 6 patients with coxarthrosis (3 females, 3 males, mean age: 66 years) undergoing hip replacement.

### 2.2. Isolation of Human CD34+ Cells

Bones were broken into fragments and incubated for 1 hour at 37 °C in phosphate-buffered saline (PBS) supplemented with 1 mg/mL collagenase type I (Thermo Fisher Scientific, Waltham, MA, USA). Supernatants were filtered in a cell strainer with 100 µm nylon mesh pores (Greiner Bio-One, Kremsmünster, Austria). The filtrates were used for CD34+ cell isolation by Ficoll density gradient centrifugation and magnetic-activated cell sorting using CD34 antibody-conjugated microbeads according to manufacturer’s protocol (Miltenyi Biotec, Bergisch Gladbach, Germany).

### 2.3. Culture of CD34+ Cells

CD34+ cells were grown in StemSpan SFEM II medium (Stemcell Technologies, Vancouver, BC, Canada) supplemented with StemSpan Myeloid Expansion supplement (SCF, TPO, G-CSF, GM-CSF) (Stemcell Technologies) and 1% penicillin/streptomycin in a humidified 5% CO_2_ atmosphere at 37 °C. CD34+ cells were grown for 3 days in untreated medium followed by culture for 3 days in medium containing the human recombinant proteins GRP78 (#NBC-118378, Novus biologicals, Littleton, CO, USA), CALR (#NBP1-44499, Novus biologicals, Littleton, CO, USA), PDIA3 (#15922189, Thermo Fisher Scientific, Waltham, MA, USA) and GPI (#SAE0005, Merck KGaA, Darmstadt, Germany), respectively. All proteins were used at a final concentration of 500 pg/µL (equivalent to concentrations of [6.4–10.6 nM] according to molecular weight). CD34+ cells grown in control medium (for 6 days) not containing any of the recombinant proteins were used as control.

### 2.4. Analysis of Genetic Instability in CD34+ Cells

Genetic instability was analyzed in CD34+ cells by immunofluorescence staining of DNA double-strand-breaks (DSB), immunofluorescence staining of centrosomes and cytogenetic analysis in CD34+ cells at day 6.

Immunofluorescence staining of DSB was performed in 1 × 10^5^ CD34+ cells using a JBW301 mouse monoclonal anti-γH2AX antibody (1:500) (#05-636, Merck KGaA) and an Alexa Fluor 488-conjugated goat anti-mouse secondary antibody (1:500) (#A11001, Thermo Fisher Scientific). At least 50 nuclei were analyzed in each sample.

Immunofluorescence staining of centrosomes and mitotic spindles was performed in 1 × 10^5^ CD34+ cells using a polyclonal anti-pericentrin (1:1000) (#ab4448, Abcam, Cambridge, UK) and a monoclonal anti-α-tubulin (1:500) (#T6074, Sigma-Aldrich, St. Louis, MO, USA) antibody as well as an Alexa Fluor 555-conjugated donkey anti-rabbit (1:1000) (#A31572, Thermo Fisher scientific) and an Alexa Fluor 488-conjugated goat anti-mouse secondary antibody (1:500) (#A11001, Thermo Fisher Scientific). At least 50 nuclei were analyzed in each sample. Centrosomes displaying > 2 centrioles were classified as numerical aberrant and centrosomes with irregular form as structural aberrant.

Cytogenetic analysis of G-banded chromosomes was performed in CD34+ cells according to standard procedures [46]. At least 25 metaphases were analyzed in each sample according to ISCN 2020 [47].

### 2.5. Statistical Analysis

Statistical calculations were carried out with SAS software, release 9.4 (SAS Institute, Cary, NC, USA). Kruskal–Wallis and exact Wilcoxon two-sample tests were performed in order to compare three or two groups, respectively. Events in CD34+ cells after exposure to recombinant GRP78, CALR, PDIA3 and GPI, respectively, were considered as independent from each other. Because of the rather small sample sizes, no correction for multiple testing has been conducted. In order to analyze the number of γH2AX foci (which may be considered as count data), Poisson regression has been used together with Dunnett’s post hoc tests for pairwise comparisons with the control group.

## 3. Results

Genotoxic effects in human CD34+ cells were analyzed after 3 days of exposure to nanomolar concentrations of recombinant GRP78, CALR, PDIA3 and GPI, respectively. Nanomolar concentrations were used in order to analyze if very low and potentially physiologically occurring concentrations of GRP78, CALR, PDIA3 and GPI induce genetic instability in CD34+ cells. The exposure time of 3 days was chosen in order to provide enough time for up to 3 cell divisions of CD34+ cells (doubling time is about 24 h) so that cytogenetics as well as centrosomes and γH2AX foci could be analyzed efficiently.

### 3.1. DNA Damage in CD34+ Cells

**Figure 1:**
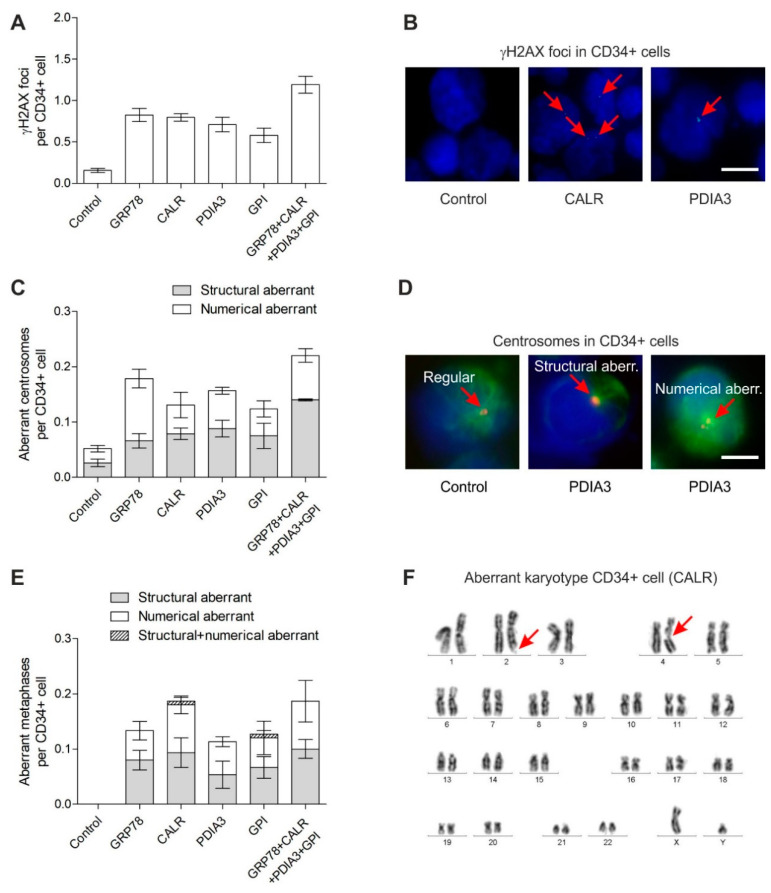
Genetic instability in CD34+ cells grown for 3 days in medium supplemented with recombinant GRP78, CALR, PDIA3 and GPI. (**A**) γH2AX foci levels in CD34+ cells (*n* = 4 patients); Poisson regression: *p* = 0.0344; Dunnett’s test each *p* < 0.0001). (**B**) Exemplary immunofluorescence images of γH2AX foci (green, Alexa 488) in nuclei (blue, DAPI) of CD34+ cells. Scale bar, 7.5 µm. (**C**) Numbers of aberrant centrosomes per CD34+ cell (*n* = 3 patients); Kruskal–Wallis test: *p* = 0.0249; Wilcoxon two-sample test: each *p* = 0.1000). (**D**) Exemplary immunofluorescence images of regular, structural aberrant and numerical aberrant centrosomes (orange, Alexa 555) and microtubules (green, Alexa 488) in nuclei (blue, DAPI) of CD34+ cells. Scale bar, 5 µm. (**E**) Numbers of aberrant metaphases per CD34+ cell (*n* = 6 patients); Kruskal–Wallis test: *p* = 0.0028; Wilcoxon two-sample test: each *p* = 0.0022. (**F**) Exemplary aberrant karyotype of a CD34+ cell grown in medium supplemented with recombinant CALR. Arrows point at chromatid breaks chtb(2q) and chtb(4q), respectively. Data in (**A** + **C** + **E**) are presented as means ± SEM.

γH2AX foci were analyzed in CD34+ cells (*n* = 4 patients) expanded for 3 days in native medium followed by culture for 3 days in medium supplemented with recombinant GRP78, CALR, PDIA3 and GPI, respectively, or in control medium. The concentration for GRP78 of 6.4 nM in our experiments was lower than a measured concentration for GRP78 of 40 nM in the serum of metastatic colorectal cancer patients [48]. γH2AX foci levels were increased about 4–5 fold in CD34+ cells grown in medium supplemented with the recombinant GRP78, CALR, PDIA3 and GPI proteins when compared to γH2AX foci levels in CD34+ cells grown in control medium (Figure 1A,B) (Poisson regression: *p* = 0.0344, Dunnett’s tests: each *p* < 0.0001). The highest γH2AX foci levels were found in CD34+ cells grown in medium supplemented with all four recombinant proteins.

### 3.2. Centrosome Aberrations in CD34+ Cells

Analogously to the γH2AX foci analysis, the centrosome aberrations were analyzed in CD34+ cells (*n* = 3 patients) after exposure to medium supplemented with the recombinant GRP78, CALR, PDIA3 and GPI proteins. Again, the numerical and structural aberrant centrosomes were found increased about 3–4 fold in CD34+ cells grown in medium supplemented with recombinant GRP78, CALR, PDIA3 and GPI when compared to aberrant centrosomes in CD34+ cells grown in control medium (Figure 1C,D) (Kruskal–Wallis test: *p* = 0.0249; Wilcoxon two-sample test: each *p* = 0.1000; *p* is not significant due to the small sample size but differences between Control and the other groups are maximal). CD34+ cells grown in medium supplemented with all four recombinant proteins demonstrated the highest level of centrosome aberrations.

### 3.3. Chromosomal Instability in CD34+ Cells

Finally, in order to test whether increased γH2AX foci and centrosome aberrations translate into chromosomal instability, the metaphases were analyzed in CD34+ cells (*n* = 6 patients) grown in medium supplemented with recombinant GRP78, CALR, PDIA3 and GPI (Figure 1E,F; Table 1). MDS/AML-associated cytogenetic alterations were detected in CD34+ cells exposed to the recombinant proteins such as tetraploidies, loss/gain of chromosomes such as −5, +8, +11, −17, +19, +21, chromatid breaks such as chtb(7q) and chtb(5q), chromosome breaks such as chsb(5q) and one translocation t(5;18). The detection of only one translocation across all metaphases is in accordance with the low frequency of translocation formation after induction of DSB [49]. Overall, aberrant metaphases were significantly increased (Kruskal–Wallis test: *p* = 0.0028; Wilcoxon two-sample test: each *p* = 0.0022) in CD34+ cells grown in medium supplemented with recombinant GRP78, CALR, PDIA3 and GPI when compared to normal metaphases in CD34+ cells grown in control medium. Particularly, high numbers of aberrant metaphases were found in CD34+ cells grown in medium supplemented with all four recombinant proteins.

## 4. Discussion

The aim of our study was to investigate potential genotoxic effects of GRP78, CALR, PDIA3 and GPI on human CD34+ cells. These proteins were previously identified as key factors in irradiated MSC conditioned medium which mediates genetic instability in CD34+ cells [7]. For this purpose, CD34+ cells were exposed for 3 days to nanomolar, i.e., potentially physiologically occurring concentrations of (recombinant) GRP78, CALR, PDIA3 and GPI and genotoxic effects were analyzed. γH2AX foci, aberrant centrosomes and aberrant metaphases were increased in CD34+ cells exposed to recombinant GRP78, CALR, PDIA3 and GPI. These characteristics do not only mark genetic instability in CD34+ cells but may indicate malignant transformation: (i) γH2AX foci increase across the spectrum from myelodysplastic syndromes (MDS) to AML [50], (ii) centrosome aberrations occur during transformation of MDS [51] and correlate with the cytogenetic risk profile of AML [52] and (iii) complex aberrant karyotypes are an adverse prognostic factor in AML [53]. 

Increased numbers of γH2AX foci indicate elevated DNA damage or defective DNA repair potentially resulting in the formation of structural chromosomal aberrations such as chromatid breaks and rarely translocations [49]. In contrast, the aberrant centrosomes indicate defective mitosis, which may result in the formation of numerical chromosomal aberrations such as tetraploidies and octaploidies. In this context, chromosomal non-disjunction and cytokinesis failure may play an important role [54]. Notably, tetraploidies are hallmark precursor lesions in diverse cancers including AML [55].

Several routes to genetic instability in CD34+ cells mediated by GRP78, CALR, PDIA3 and GPI are conceivable. Clearly, the re-localized and secreted proteins exert non-canonical (oncogenic) functions that differ from their canonical (physiological) functions in their regular intracellular compartment.

As a cell surface receptor, GRP78 may activate PI3K/AKT oncogenic signaling in CD34+ cells similar to its role in Pten-null driven leukemogenesis [56]. PI3K/AKT may override cell cycle checkpoints [57], inhibit DSB repair [58] and abrogate apoptosis [59]. Further, PI3K may promote centrosome amplification [60,61] and prolong microtubule stabilization [62] causing chromosomal instability. Additional evidence for a role of GRP78 in centrosome dynamics comes from the Drosophila homolog Hsc70-3, which has been reported to be involved in centrosome duplication and segregation [63]. CALR might affect hematopoietic stem cell differentiation by its interference with ER stress, the unfolded protein response and DNA repair [64]. Further, CALR is a major Ca^2+^ storage protein, which accumulates in the pericentriolar region upon proteasomal inhibition [65]. Here, CALR may perturb Ca^2+^ signaling from the ER which is essential for mitosis [66]. PDIA3 is another critical chaperone in the ER that was found to be essential for H2AX phosphorylation in response to chemotherapy-induced DNA damage [67]. From the lumen of the ER, PDIA3 may modulate oncogenic STAT3 signaling which may cause genomic instability by inhibition of apoptosis and overriding of cell-cycle checkpoints [38]. Further, PDIA3 may activate metalloproteases and integrins on the surface of CD34+ cells involved in anti-apoptotic, pro-migratory and pro-mitogenic signaling [39,68]. Lastly, the glycolytic enzyme GPI might induce genetic instability in CD34+ cells by activation of MAPK/ERK [43] and PI3K/AKT [44] signaling pathways. Our study has some limitations that need to be addressed. (i) Recombinant proteins may differ from endogenous proteins in specific properties such as posttranslational modifications, tertiary and quaternary structures. Therefore, our experiments in vitro naturally differ from signaling between irradiated MSC and CD34+ cells in vivo. (ii) Our experiments were performed without control proteins such as β-Actin, GUSB or GAPDH, but the control medium contained albumin, insulin, transferrin as well as recombinant SCF, TPO, G-CSF and GM-CSF that demonstrated no adverse effects on genomic stability. (iii) GRP78, CALR, PDIA3 and GPI represent a snapshot in the secretome of irradiated MSC, whereas the secretion of factors is a dynamic process and other factors might be relevant at different times as well. Finally, we would like to emphasize the medical impact of studying genotoxic signaling by irradiated MSC. MSC play a critical role in the haematopoietic stem cell (HSC) niche and are necessary in regulating HSC self-renewal and function. The release of genotoxic factors by irradiated MSC into the microenvironment may interfere with HSC function and potentially contribute to leukemogenesis. Furthermore, as MSC are relatively radioresistant, they may survive radiation doses such as those applied during total body irradiation for allogeneic bone marrow transplantation, whereas radio-sensitive HSC are rapidly depleted [69,70]. Consequently, they may interfere with remaining or transplanted HSC. Overall, a better understanding of the underlying mechanisms may not only improve our knowledge about radiation-induced pathogenic bone marrow conditions but also contribute to the development of therapeutic strategies.

## 5. Conclusions

Our data suggest that GRP78, CALR, PDIA3 and GPI released from irradiated MSC act as mediators of genetic instability in human CD34+ cells. These findings provide critical insights into the development of radiation-induced hematologic disorders with potential implications for therapeutic interventions.

## Figures and Tables

**Table 1:** Cytogenetics in CD34+ cells grown for 3 days in medium supplemented with recombinant GRP78, CALR, PDIA3 and GPI. chsb, chromosome break; chtb, chromatid break; ISCN, international system for human cytogenetic nomenclature; min, minute (acentric fragment smaller than the width of a single chromatid); f, fragment; Pt, patient; [number], number of analyzed metaphases.

Pt	Age/ Sex	Cytogenetics (ISCN) CD34^+^ Cells					
		Control	+GRP78	+CALR	+PDIA3	+GPI	+GRP78 + CALR +PDIA3 + GPI
#1	78/♀	46,XX[25]	46,XX[23] 46,XX,chtb(2q)[1] 92,XXXX[1]	46,XX[20] 46,XX,chtb(Xq)[1] 46,XX,+min[1] 47,XX,+14[1] 92,XXXX[2]	46,XX[21] 46,XX,chtb(5q)[1] 46,XX,chtb(7p), +min[1] 46,XX,chtb(10q)[1] 47,XX,+21[1]	46,XX[21] 46,XX,chtb(3q)[1] 46,XX,chtb(4q)[1] 46,XX,chtb(10q)[1] 47,XX,+15[1]	46,XX[20] 46,XX,chtb(4q)[1] 92,XXXX[4]
#2	71/♂	46,XY[25]	46,XY[22] 46,XY,chtb(5q)[1] 46,XY,chtb(18q)[1] 54,XXY,+6,+7,+9, +12,+16,+17,+18[1]	46,XY[19] 46,XY,chtb(2q), chtb(4q)[1] 47,XY,+2, chtb(2q)[1] 47,XY,+1[1] 46,XY,+min[1] 69,XXY[2]	46,XY[23] 92,XXYY[2]	46,XY[21] 46,XY,chtb(10q)[1] 45,XY,-5[1] 46,XY,chtb(7q), chtb(18q)[1] 45,XY,der(5)t(5;18) (q35;q23)[1]	46,XY[20] 46,XY,chtb(2q)[2] 46,XY,chtb(16q)[1] 47,XY,+8[1] 46,XY,chtb(1p)[1]
#3	44/♂	46,XY[25]	46,XY[21] 46,XY,chtb(2q)[1] 46,XY,chtb(4q)[1] 46,XY,chtb(15q)[1] 46,XY,chtb(5q)[1]	46,XY[22] 45,XY,-5[1] 46,XY,chtb(14q)[1] 46,XY,chtb(15q)[1]	46,XY[21] 45,XY,chtb(20p)[1] 46,XY,chtb(5q)[1] 45,XY,-5[1] 92,XXYY[1]	46,XY[23] 45,XY,-14,-17,+f[1] 46,XY,chtb(8q)[1]	46,XY[22] 45,XY,-5[1] 46,XY,chtb(10q)[1] 46,XY,chtb(3q)[1]
#4	78/♀	46,XX[25]	46,XX[21] 46,XX,chtb(2q)[1] 47,XX,+8[1] 47,XX,+20[1] 48,XX,+8,+20[1]	46,XX[20] 46,XX,chtb(1p)[1] 46,XX,chtb(15q)[1] 46,XX,chtb(7p)[1] 50,XX,+1,+6,+11, +17[1] 92,XXXX[1]	46,XX[21] 46,XX, del(7)(p11)[1] 46,XX,chtb(1q)[1] 46,XX,chtb(7q)[1] 47,XX,+18[1]	46,XX[23] 45,XX,chtb(4q)[1] 46,XX,chtb(16q)[1]	46,XX[16] 92,XXXX[5] 47,XX,+16[1] 46,XX,chtb(4q)[1] 46,XX,chtb(10q)[1] 46,XX,chtb(9q)[1]
#5	53/♂	46,XY[25]	46,XY[22] 47,XY,+12[1] 46,XY,chtb(7q)[1] 46,XY,chsb(5q)[1]	46,XY[24] 47,XY,+19[1]	46,XY[23] 92,XXYY[1] 184,XXXXYYYY[1]	46,XY[24] 47,XY,+9[1]	46,XY[23] 46,XY,chtb(7q)[1] 46,XY,chtb(12q)[1]
#6	73/♀	46,XX[25]	46,XX[21] 46,XX,chtb(14q)[1] 46,XX,chtb(Xq)[1] 47,XX,+9[1] 47,XX,+14[1]	46,XX[17] 47,XX,+9[1] 46,XX,chsb(3p)[1] 46,XX,chtb(5p)[2] 46,XX,chtb(4q)[2] 92,XXXX[2]	46,XX[24] 92,XXXX[1]	46,XX[19] 46,XX,chtb(8q)[1] 48,XX,+3,+16[1] 92,XXXX[3] 184,XXXXXXXX[1]	46,XX[21] 46,XX,chtb(8q)[1] 46,XX,chtb(13q)[1] 47,XX,+f[1] 92,XXXX[1]

## Data Availability

The data that support the findings of this study are available from the corresponding author upon reasonable request.

## References

[1] Hu L., Yin X., Zhang Y., Pang A., Xie X., Yang S., Zhu C., Li Y., Zhang B., Huang Y. (2021). Radiation-induced bystander effects impair transplanted human hematopoietic stem cells via oxidative DNA damage. Blood.

[2] Mothersill C., Rusin A., Seymour C. (2019). Relevance of Non-Targeted Effects for Radiotherapy and Diagnostic Radiology; A Historical and Conceptual Analysis of Key Players. Cancers.

[3] Nikitaki Z., Mavragani I.V., Laskaratou D.A., Gika V., Moskvin V.P., Theofilatos K., Vougas K., Stewart R.D., Georgakilas A.G. (2016). Systemic mechanisms and effects of ionizing radiation: A new ‘old’ paradigm of how the bystanders and distant can become the players. Semin. Cancer Biol..

[4] Heeran A.B., Berrigan H.P., O’Sullivan J. (2019). The Radiation-Induced Bystander Effect (RIBE) and its Connections with the Hallmarks of Cancer. Radiat. Res..

[5] Lorimore S.A., McIlrath J.M., Coates P.J., Wright E.G. (2005). Chromosomal instability in unirradiated hemopoietic cells resulting from a delayed in vivo bystander effect of gamma radiation. Cancer Res..

[6] Lorimore S.A., Chrystal J.A., Robinson J.I., Coates P.J., Wright E.G. (2008). Chromosomal instability in unirradiated hemaopoietic cells induced by macrophages exposed in vivo to ionizing radiation. Cancer Res..

[7] Kohl V., Drews O., Costina V., Bierbaum M., Jawhar A., Roehl H., Weiss C., Brendel S., Kleiner H., Flach J. (2021). Proteins Marking the Sequence of Genotoxic Signaling from Irradiated Mesenchymal Stromal Cells to CD34+ Cells. Int. J. Mol. Sci..

[8] Kohl V., Fabarius A., Drews O., Bierbaum M., Jawhar A., Darwich A., Weiss C., Flach J., Brendel S., Kleiner H. (2021). Genotoxic Bystander Signals from Irradiated Human Mesenchymal Stromal Cells Mainly Localize in the 10-100 kDa Fraction of Conditioned Medium. Cells.

[9] Chen S., Zhao Y., Han W., Zhao G., Zhu L., Wang J., Bao L., Jiang E., Xu A., Hei T.K. (2008). Mitochondria-dependent signalling pathway are involved in the early process of radiation-induced bystander effects. Br. J. Cancer.

[10] Lyng F.M., Howe O.L., McClean B. (2011). Reactive oxygen species-induced release of signalling factors in irradiated cells triggers membrane signalling and calcium influx in bystander cells. Int. J. Radiat. Biol..

[11] Tartier L., Gilchrist S., Burdak-Rothkamm S., Folkard M., Prise K.M. (2007). Cytoplasmic irradiation induces mitochondrial-dependent 53BP1 protein relocalization in irradiated and bystander cells. Cancer Res..

[12] Shao C., Stewart V., Folkard M., Michael B.D., Prise K.M. (2003). Nitric oxide-mediated signaling in the bystander response of individually targeted glioma cells. Cancer Res..

[13] Jella K.K., Moriarty R., McClean B., Byrne H.J., Lyng F.M. (2018). Reactive oxygen species and nitric oxide signaling in bystander cells. PLoS ONE.

[14] Li J., He M., Shen B., Yuan D., Shao C. (2013). Alpha particle-induced bystander effect is mediated by ROS via a p53-dependent SCO2 pathway in hepatoma cells. Int. J. Radiat. Biol..

[15] Desai S., Kumar A., Laskar S., Pandey B.N. (2013). Cytokine profile of conditioned medium from human tumor cell lines after acute and fractionated doses of gamma radiation and its effect on survival of bystander tumor cells. Cytokine.

[16] Shao C., Folkard M., Prise K.M. (2008). Role of TGF-beta1 and nitric oxide in the bystander response of irradiated glioma cells. Oncogene.

[17] Gow M.D., Seymour C.B., Ryan L.A., Mothersill C.E. (2010). Induction of bystander response in human glioma cells using high-energy electrons: A role for TGF-beta1. Radiat. Res..

[18] Peng Y., Zhang M., Zheng L., Liang Q., Li H., Chen J.T., Guo H., Yoshina S., Chen Y.Z., Zhao X. (2017). Cysteine protease cathepsin B mediates radiation-induced bystander effects. Nature.

[19] Zhou H., Ivanov V.N., Lien Y.C., Davidson M., Hei T.K. (2008). Mitochondrial function and nuclear factor-kappaB-mediated signaling in radiation-induced bystander effects. Cancer Res..

[20] Lyng F.M., Maguire P., McClean B., Seymour C., Mothersill C. (2006). The involvement of calcium and MAP kinase signaling pathways in the production of radiation-induced bystander effects. Radiat. Res..

[21] Xu S., Wang J., Ding N., Hu W., Zhang X., Wang B., Hua J., Wei W., Zhu Q. (2015). Exosome-mediated microRNA transfer plays a role in radiation-induced bystander effect. RNA Biol..

[22] Ariyoshi K., Miura T., Kasai K., Fujishima Y., Nakata A., Yoshida M. (2019). Radiation-Induced Bystander Effect is Mediated by Mitochondrial DNA in Exosome-Like Vesicles. Sci. Rep..

[23] Kirolikar S., Prasannan P., Raghuram G.V., Pancholi N., Saha T., Tidke P., Chaudhari P., Shaikh A., Rane B., Pandey R. (2018). Prevention of radiation-induced bystander effects by agents that inactivate cell-free chromatin released from irradiated dying cells. Cell Death Dis..

[24] Shao C., Furusawa Y., Aoki M., Ando K. (2003). Role of gap junctional intercellular communication in radiation-induced bystander effects in human fibroblasts. Radiat. Res..

[25] Hetz C., Zhang K., Kaufman R.J. (2020). Mechanisms, regulation and functions of the unfolded protein response. Nat. Rev. Mol. Cell Biol..

[26] Meusser B., Hirsch C., Jarosch E., Sommer T. (2005). ERAD: The long road to destruction. Nat. Cell Biol..

[27] Ni M., Zhang Y., Lee A.S. (2011). Beyond the endoplasmic reticulum: Atypical GRP78 in cell viability, signalling and therapeutic targeting. Biochem. J..

[28] Kern J., Untergasser G., Zenzmaier C., Sarg B., Gastl G., Gunsilius E., Steurer M. (2009). GRP-78 secreted by tumor cells blocks the antiangiogenic activity of bortezomib. Blood.

[29] Kelber J.A., Panopoulos A.D., Shani G., Booker E.C., Belmonte J.C., Vale W.W., Gray P.C. (2009). Blockade of Cripto binding to cell surface GRP78 inhibits oncogenic Cripto signaling via MAPK/PI3K and Smad2/3 pathways. Oncogene.

[30] Staquicini D.I., D’Angelo S., Ferrara F., Karjalainen K., Sharma G., Smith T.L., Tarleton C.A., Jaalouk D.E., Kuniyasu A., Baze W.B. (2018). Therapeutic targeting of membrane-associated GRP78 in leukemia and lymphoma: Preclinical efficacy in vitro and formal toxicity study of BMTP-78 in rodents and primates. Pharm. J..

[31] Hebbar N., Epperly R., Vaidya A., Thanekar U., Moore S.E., Umeda M., Ma J., Patil S.L., Langfitt D., Huang S. (2022). CAR T cells redirected to cell surface GRP78 display robust anti-acute myeloid leukemia activity and do not target hematopoietic progenitor cells. Nat. Commun..

[32] Fucikova J., Spisek R., Kroemer G., Galluzzi L. (2020). Calreticulin and cancer. Cell Res..

[33] Obeid M., Tesniere A., Ghiringhelli F., Fimia G.M., Apetoh L., Perfettini J.L., Castedo M., Mignot G., Panaretakis T., Casares N. (2007). Calreticulin exposure dictates the immunogenicity of cancer cell death. Nat. Med..

[34] Prins D., Gonzalez Arias C., Klampfl T., Grinfeld J., Green A.R. (2020). Mutant Calreticulin in the Myeloproliferative Neoplasms. Hemasphere.

[35] Bourdi M., Demady D., Martin J.L., Jabbour S.K., Martin B.M., George J.W., Pohl L.R. (1995). cDNA cloning and baculovirus expression of the human liver endoplasmic reticulum P58: Characterization as a protein disulfide isomerase isoform, but not as a protease or a carnitine acyltransferase. Arch. Biochem. Biophys..

[36] Oliver J.D., van der Wal F.J., Bulleid N.J., High S. (1997). Interaction of the thiol-dependent reductase ERp57 with nascent glycoproteins. Science.

[37] Chichiarelli S., Altieri F., Paglia G., Rubini E., Minacori M., Eufemi M. (2022). ERp57/PDIA3: New insight. Cell Mol. Biol. Lett..

[38] Coe H., Jung J., Groenendyk J., Prins D., Michalak M. (2010). ERp57 modulates STAT3 signaling from the lumen of the endoplasmic reticulum. J. Biol. Chem..

[39] Lee E., Lee D.H. (2017). Emerging roles of protein disulfide isomerase in cancer. BMB Rep..

[40] Ye Q., Fu P., Dou J., Wang N. (2018). Downregulation of PDIA3 inhibits proliferation and invasion of human acute myeloid leukemia cells. Onco Targets Ther..

[41] Funasaka T., Haga A., Raz A., Nagase H. (2001). Tumor autocrine motility factor is an angiogenic factor that stimulates endothelial cell motility. Biochem. Biophys. Res. Commun..

[42] Tsutsumi S., Gupta S.K., Hogan V., Collard J.G., Raz A. (2002). Activation of small GTPase Rho is required for autocrine motility factor signaling. Cancer Res..

[43] Araki K., Shimura T., Yajima T., Tsutsumi S., Suzuki H., Okada K., Kobayashi T., Raz A., Kuwano H. (2009). Phosphoglucose isomerase/autocrine motility factor promotes melanoma cell migration through ERK activation dependent on autocrine production of interleukin-8. J. Biol. Chem..

[44] Tsutsumi S., Hogan V., Nabi I.R., Raz A. (2003). Overexpression of the autocrine motility factor/phosphoglucose isomerase induces transformation and survival of NIH-3T3 fibroblasts. Cancer Res..

[45] Fu M., Li L., Albrecht T., Johnson J.D., Kojic L.D., Nabi I.R. (2011). Autocrine motility factor/phosphoglucose isomerase regulates ER stress and cell death through control of ER calcium release. Cell Death Differ..

[46] Heim S., Mitelman F. (2009). Cancer Cytogenetics.

[47] McGowan-Jordan J., Hastings R.J., Moore S. (2020). An International System for Human Cytogenetic Nomenclature.

[48] La X., Zhang L., Li H., Li Z., Song G., Yang P., Yang Y. (2018). Ajuba receptor mediates the internalization of tumor-secreted GRP78 into macrophages through different endocytosis pathways. Oncotarget.

[49] Roukos V., Voss T.C., Schmidt C.K., Lee S., Wangsa D., Misteli T. (2013). Spatial dynamics of chromosome translocations in living cells. Science.

[50] Popp H.D., Naumann N., Brendel S., Henzler T., Weiss C., Hofmann W.K., Fabarius A. (2017). Increase of DNA damage and alteration of the DNA damage response in myelodysplastic syndromes and acute myeloid leukemias. Leuk Res..

[51] Ruppenthal S., Kleiner H., Nolte F., Fabarius A., Hofmann W.K., Nowak D., Seifarth W. (2018). Increased separase activity and occurrence of centrosome aberrations concur with transformation of MDS. PLoS ONE.

[52] Neben K., Giesecke C., Schweizer S., Ho A.D., Kramer A. (2003). Centrosome aberrations in acute myeloid leukemia are correlated with cytogenetic risk profile. Blood.

[53] Stolzel F., Mohr B., Kramer M., Oelschlagel U., Bochtler T., Berdel W.E., Kaufmann M., Baldus C.D., Schafer-Eckart K., Stuhlmann R. (2016). Karyotype complexity and prognosis in acute myeloid leukemia. Blood Cancer J..

[54] Tanaka K., Goto H., Nishimura Y., Kasahara K., Mizoguchi A., Inagaki M. (2018). Tetraploidy in cancer and its possible link to aging. Cancer Sci..

[55] Huang L., Wang S.A., DiNardo C., Li S., Hu S., Xu J., Zhou W., Goswami M., Medeiros L.J., Tang G. (2017). Tetraploidy/near-tetraploidy acute myeloid leukemia. Leuk Res..

[56] Wey S., Luo B., Tseng C.C., Ni M., Zhou H., Fu Y., Bhojwani D., Carroll W.L., Lee A.S. (2012). Inducible knockout of GRP78/BiP in the hematopoietic system suppresses Pten-null leukemogenesis and AKT oncogenic signaling. Blood.

[57] Henry M.K., Lynch J.T., Eapen A.K., Quelle F.W. (2001). DNA damage-induced cell-cycle arrest of hematopoietic cells is overridden by activation of the PI-3 kinase/Akt signaling pathway. Blood.

[58] Plo I., Laulier C., Gauthier L., Lebrun F., Calvo F., Lopez B.S. (2008). AKT1 inhibits homologous recombination by inducing cytoplasmic retention of BRCA1 and RAD51. Cancer Res..

[59] Tonic I., Yu W.N., Park Y., Chen C.C., Hay N. (2010). Akt activation emulates Chk1 inhibition and Bcl2 overexpression and abrogates G2 cell cycle checkpoint by inhibiting BRCA1 foci. J. Biol. Chem..

[60] Nam H.J., Chae S., Jang S.H., Cho H., Lee J.H. (2010). The PI3K-Akt mediates oncogenic Met-induced centrosome amplification and chromosome instability. Carcinogenesis.

[61] Berenjeno I.M., Pineiro R., Castillo S.D., Pearce W., McGranahan N., Dewhurst S.M., Meniel V., Birkbak N.J., Lau E., Sansregret L. (2017). Oncogenic PIK3CA induces centrosome amplification and tolerance to genome doubling. Nat. Commun..

[62] Onishi K., Higuchi M., Asakura T., Masuyama N., Gotoh Y. (2007). The PI3K-Akt pathway promotes microtubule stabilization in migrating fibroblasts. Genes Cells.

[63] Muller H., Schmidt D., Steinbrink S., Mirgorodskaya E., Lehmann V., Habermann K., Dreher F., Gustavsson N., Kessler T., Lehrach H. (2010). Proteomic and functional analysis of the mitotic Drosophila centrosome. EMBO J..

[64] Salati S., Prudente Z., Genovese E., Pennucci V., Rontauroli S., Bartalucci N., Mannarelli C., Ruberti S., Zini R., Rossi C. (2018). Calreticulin Affects Hematopoietic Stem/Progenitor Cell Fate by Impacting Erythroid and Megakaryocytic Differentiation. Stem. Cells Dev..

[65] Kamhi-Nesher S., Shenkman M., Tolchinsky S., Fromm S.V., Ehrlich R., Lederkremer G.Z. (2001). A novel quality control compartment derived from the endoplasmic reticulum. Mol. Biol. Cell.

[66] Helassa N., Nugues C., Rajamanoharan D., Burgoyne R.D., Haynes L.P. (2019). A centrosome-localized calcium signal is essential for mammalian cell mitosis. FASEB J..

[67] Krynetskaia N.F., Phadke M.S., Jadhav S.H., Krynetskiy E.Y. (2009). Chromatin-associated proteins HMGB1/2 and PDIA3 trigger cellular response to chemotherapy-induced DNA damage. Mol. Cancer Ther..

[68] Cooper J., Giancotti F.G. (2019). Integrin Signaling in Cancer: Mechanotransduction, Stemness, Epithelial Plasticity, and Therapeutic Resistance. Cancer Cell.

[69] Nicolay N.H., Lopez Perez R., Saffrich R., Huber P.E. (2015). Radio-resistant mesenchymal stem cells: Mechanisms of resistance and potential implications for the clinic. Oncotarget.

[70] Sugrue T., Lowndes N.F., Ceredig R. (2013). Mesenchymal stromal cells: Radio-resistant members of the bone marrow. Immunol. Cell Biol..

